# The Risk Status of Waiting Areas for Airborne Infection Control in Delhi Hospitals

**DOI:** 10.7759/cureus.23211

**Published:** 2022-03-16

**Authors:** Raja Singh

**Affiliations:** 1 Architecture, School of Planning and Architecture, New Delhi, IND

**Keywords:** high incidence of tuberculosis, infection prevention and control, crowdedness, nosocomial infection, infection control measures, airborne infection control, waiting areas

## Abstract

Background

Hospital waiting areas are overlooked from the airborne infection control viewpoint as they are not classified as critical for infection control. This is the area where undiagnosed and potentially infected patients gather with susceptible and vulnerable patients, and there is no mechanism to segregate the two, especially when the potentially infected visitors/patients themselves are unaware of the infection or may be asymptomatic. It is important to know whether hospitals in Delhi, a populated, low-resource setting having community transmission/occurrence of airborne diseases such as tuberculosis, consider waiting areas as critical. Hence, this study aims to determine whether hospitals in Delhi consider waiting areas as critical areas from the airborne infection control viewpoint.

Methodology

The Right to Information Act, 2005, was used to request information from 11 hospitals included in this study.

Results

After compiling the results, it was found that five out of the 11 hospitals did not consider waiting areas as critical from the infection spread point of view. Two of the 11 hospitals acknowledged the criticality of waiting areas but did not include the same in the list of critical areas. Only three out of the 11 considered waiting areas as critical and included these in the list of critical areas in a hospital.

Conclusions

This study provided evidence that most hospitals in Delhi do not include waiting areas in the list of critical areas in a hospital.

## Introduction

Almost all hospitals have hospital infection control committees that have a set procedure for enabling measures to prevent the spread of nosocomial infections. This involves infection transmission from patients to healthcare workers. This procedure involves surveillance measures and routine checks through various methods in various parts of the hospitals. There is a usual list of areas that the hospital infection committee takes special care of and these areas are called critical or high-risk areas. There can be multiple reasons why an area can be considered a high-risk area, but the most important reason is the presence of susceptible individuals and those with infections. This means transmission of infection from patients to healthcare workers, patients, and visitors. Under the category of areas with the most susceptible people, hospitals usually categorize intensive care units, high-dependency units, operation theaters, nurseries, and labor rooms as usual high-risk or critical areas. This approach is appropriate as it focuses on saving the most vulnerable. However, there is another approach of preventing the most infectious individuals to contact people or areas where susceptible patients or others are present. This is all the more true for diseases where the infection spreads through the airborne route. The Indian guidelines for airborne prevention include measures such as patient segregation, which differentiates between respiratory patients with symptoms and directs their movement through a different channel [1]. It also suggests measures such as dilution ventilation to prevent the spread of infection. This approach has also been followed by other studies aiming to reduce potential opportunities of exposure to susceptible individuals by patient segregation [2]. However, this approach may not be enough as there are patients who turn up to a hospital for the first time and have not been diagnosed. The areas that they usually stay for a longer time before being screened are the waiting areas of hospitals. This is all the more true for healthcare settings in developing countries which are usually overcrowded [3]. A previously reported flow analysis of patients showed that any person visiting the emergency department and having an infectious pathogen may first pass through a triage area. This area may have appropriate infection control but spills into the waiting area where there are attendants and sometimes other patients present. Hence, waiting areas should be considered high-risk areas [4]. This fact has also been highlighted by another study which stated that certain spaces in the hospital such as patient areas and procedural rooms are pressurized to control airflow, thus limiting the airborne spread of infection. The study further stated that other areas are overlooked and point to waiting areas where healthy people gather, stay, and contact infected people directly and indirectly [5]. The fact that waiting areas need to be paid attention to from the airborne infection spread viewpoint has also been concurred by other studies [6,7]. However, a notable study also questioned the implementation of airborne infection control measures in waiting areas but included a disclaimer that this may not be true for developing countries with overcrowded waiting halls [8]. The waiting areas become all the more critical due to the possibility of spread of airborne diseases from asymptomatic patients who spread the disease due to a lack of diagnosis. The position that Indian waiting halls are crowded and need to be classified as critical or high-risk areas is getting strengthened in study after study.

This study examines hospitals in New Delhi, the capital of India which is the world’s second most populated country. It aims to investigate the status of hospital waiting areas as to whether they are considered high risk/critical. New Delhi is India’s second most populated city after Mumbai. India has a high load of nosocomial tuberculosis which is one of the most deadly airborne diseases with occurrences of community spread. Healthcare facilities in Delhi are crowded and need additional care to prevent nosocomial airborne spread of tuberculosis and other diseases that spread through the airborne route [9-12]. The spread of coronavirus disease 2019 infection through the airborne route has also increased the risk in the waiting halls of these spaces. This is all the more true due to the asymptomatic spread and high transmissibility of new diseases [13,14]. There has been no similar study conducted with a focus on waiting areas in hospitals of Delhi, which is a low-resource setting having a high load of airborne diseases and crowded healthcare facilities.

In this study, we aimed to determine whether waiting areas are considered critical or high-risk areas in hospitals in Delhi, especially from the airborne infection spread viewpoint using open public domain information provided by the hospitals.

## Materials and methods

This study has used a unique method for obtaining information from hospitals that are generally believed to be reluctant toward sharing information [15]. In India, due to bureaucratic pressures, hospitals may be wary of sharing information, especially one related to infection spread in the hospital. This study used the Right to Information Act, 2005, as a research tool to gather information from public-owned hospitals that are designated public authorities under the said Act and are duty bound to provide the information [16,17]. Applications were filed under the Right to Information Act, 2005, to the Public Information Officers in these hospitals who are usually the heads of Infection Control Committees, Head of Microbiology Departments, Medical Superintendents, or other highly placed hospital administrators. This approach ensured accurate, legal, ethical, and certified information supply from the hospitals. The study period was the beginning phase of the declaration of the pandemic when Delhi had just seen the first case of COVID-19.

A total of 11 hospitals in Delhi were asked to share the information. Out of these 11 hospitals, seven are operated by the Delhi Government. The total number of hospitals in Delhi is 56, and the total number of hospitals run by the Delhi Government is 37 [18,19]. The sample size can be justified as follows: if taking all government hospitals in consideration has an accuracy of 85% ± 20%, and the same is 80% ± 23% when only the hospitals run by the Delhi government are considered. Only government-run hospitals can be studied using this methodology as private hospitals are not under the purview of the Right to Information Act, 2005. The list of the hospitals along with their bed capacity (as a broad indicator of their size) is provided in Table 1.

**Table 1:** List of hospitals included in this study.

Name of hospital	Remarks
Attar Sain Jain Eye & General Hospital	30-bed eye care hospital.
Chacha Nehru Bal Chikitsalaya	221-bed superspecialty pediatric hospital
Dr. Ram Manohar Lohia Hospital	1,420-bed teaching hospital
Bhagwan Mahavir Hospital	325-bed hospital
Babu Jagjivan Ram Memorial Hospital	100-bed hospital
Aruna Asaf Ali Government Hospital	100-bed hospital
Dr. Baba Saheb Ambedkar Hospital	500-bed multispecialty hospital
Acharyashree Bhikshu Government Hospital	150-bed hospital
All India Institute of Medical Sciences, New Delhi	2,362-bed, apex-level tertiary care teaching and research hospital
Hindu Rao Hospital	980-bedded multispecialty hospital
National Institute of Tuberculosis and Respiratory Diseases	National-level hospital for tuberculosis and respiratory diseases. 354-bed teaching, training, and research hospital

It must be noted that out of the above, one (All India Institute of Medical Sciences, New Delhi) is an apex, tertiary level care hospital, where top leaders of the country are treated; moreover, this hospital is a top-level, public-funded research teaching hospital. The National Institute of Tuberculosis and Respiratory Diseases is a national-level top hospital dealing with tuberculosis. Dr. Ram Manohar Lohia Hospital and Hindu Rao Hospital are considerably important hospitals with high patient interaction.

The information requested from the hospitals as an application under the Right to Information Act, 2005, is listed in Table 2.

**Table 2:** The information required from the hospitals included in the study.

Information required
Details of specific measures taken to prevent the spread of airborne infections
List of the areas that are considered critical as far as infection control is concerned
Waiting areas as critical areas. Whether they are listed as critical by the hospital

Non-requirement of ethics clearance for this study

This study included no questionnaire, and no human, including any employee, doctor, visitor, or staff, was contacted directly for this study. This study used information available in the public domain through the Right to Information Act, 2005, where an application was made under Section of the Act and the information was supplied. The information provided was signed and certified by the hospital through a senior official and released into the public domain. The Right to Information Act, 2005, allows for the provision of only such information that is not third-party or personal information of any individual. This prevents any information of any human subject. The use of information available under the public domain for any systemic review is exempt for review under the Indian Council of Medical Research, National Ethics Guidelines for Biomedical and Health Research involving Human Participants. It further goes on to state that the scope of ethics is only for studies involving human participants. According to the above-mentioned scope, this study does not require Ethics Committee Approval or its equivalent Institutional Review Board Approval [20]. The author declares the same.

## Results

The above-mentioned details were filed as applications and the replies were received. The replies received from the 11 hospitals included in this study have been compiled in Table 3. Hindu Rao Hospital and Baba Ambedkar Hospital provided the least amount of information. The All India Institute of Medical Sciences may not have provided complete information compared to the other hospitals included in the study.

**Table 3:** The compilation of the information received from the hospitals. OT: operation theater; ICU: intensive care unit; HDU: high-dependency unit; CSSD: Central Sterile Supply Unit; TB: tuberculosis; OPD: outpatient department; ART: antiretroviral therapy; DOTS: directly observed treatment, short-course; NICU: neonatal intensive care unit; PPE: personal protective equipment; IEC: information, education, and communication; HEPA: high-efficiency particulate absorbing filter

Name of hospital	Details of specific measures taken to prevent the spread of airborne infections	Areas that are considered critical as far as infection control is concerned	Whether waiting areas are considered critical	Remarks
Attar Sain Jain Eye & General Hospital	Being a dedicated daycare eye center, standard infection control precautions are taken for OT, ward, and patient waiting areas.	Critical high-risk areas: (1) OTs, (2) drinking water, (3) waiting areas	Yes. Critical from the infection spread point of view as this is the most crowded with patients and attendants	Information provided as a signed public domain copy by the Public Information Officer designated by the hospital
Chacha Nehru Bal Chikitsalaya	(1) Isolation; (2) all standard precautions in addition to the transmission-based precautions	High-risk areas: (1) ICUs, (2) OTs, (3) HDU, (4) CSSD, (5) dialysis, (6) transfusion services unit, (7) kitchen, (8) drinking water services	Waiting areas should have marks for social distancing, and IEC material to prevent infection spread	Waiting area not stated critical, nor part of the list of critical areas. Though some interventions have been stated. Information provided as a signed public domain copy by the Public Information Officer designated by the hospital
Dr. Ram Manohar Lohia Hospital	(1) Education regarding cough/respiratory etiquette; (2) preventing overcrowding; (3) following hand hygiene and standard precautions; (4) use of PPE masks in wards/OPDs dealing with airborne infection	Critical areas: (1) ICUs, (2) OTs, (3) minor OTs, (4) burns and plastic surgery. Infection control followed in all areas	Waiting areas are critical due to heavy patient footfall and overcrowding	Waiting areas stated to be crucial, but not included in the list of critical areas. Information provided as a signed public domain copy by the Public Information Officer designated by the hospital
Bhagwan Mahavir Hospital	All measures as per the Infection Control Guidelines	Critical areas: (1) ICUs, (2) NICUs, (3) OTs and minor OTs, (4) labor room	No	Information provided as a signed public domain copy by the Public Information Officer designated by the hospital
Babu Jagjivan Ram Memorial Hospital	(1) Standard precautions, (2) counseling of patients about cough hygiene/respiratory etiquette; (3) prioritization of symptomatic patients, (4) Fast tracking of symptomatic patients; (5) adequate PPE use as and when required by healthcare workers	Critical areas: (1) OTs, (2) Nursery ICU; (3) labor room	No	Information provided as a signed public domain copy by the Public Information Officer designated by the hospital
Aruna Asaf Ali Government Hospital	Wearing masks in critical areas	OT, labor room, minor OT, and casualty	Not answered	Information provided as a signed public domain copy by the Public Information Officer designated by the hospital
Dr. Baba Saheb Ambedkar Hospital	Provided the Hospital Infection Control Manual		Provided the Hospital Infection Control Manual	Information provided as a signed public domain copy by the Public Information Officer designated by the hospital
Acharyashree Bhikshu Government Hospital	(1) PPE to all healthcare workers; (2) masks provided to suspect patients; (3) decontamination of surfaces done with chemical disinfectants; (4) availability of HEPA filters in OTs	Critical areas: (1) Emergency OT, (2) main OT; (3) high-dependency unit; (4) special newborn care unit	Not considered critical. Waiting area for flu considered critical and disinfected. OT considered critical. Waiting areas and wards not considered critical. There is no ART clinic and no DOTS clinic	Information provided as a signed public domain copy by the Public Information Officer designated by the hospital
All India Institute of Medical Sciences, New Delhi	Provided the Infection Control Manual	Each healthcare facility has to define its own critical care areas depending on the disease profile of admitted patients in the facility. These may include ICUs and OTs	Information not provided (Law quoted to prevent the furnishing of information) There is no such information available in the records of the hospital	The answers may be deflecting, with a possibility of no intention of providing direct answers in a transparent manner compared to other hospitals. Information provided as a signed public domain copy by the Public Information Officer designated by the hospital
Hindu Rao Hospital	“No requisite information is available on record”	“No requisite information is available on record”	“No requisite information is available on record”	Information provided as a signed public domain copy by the Public Information Officer designated by the hospital
National Institute of Tuberculosis and Respiratory Diseases	Follow procedures as per “Guidelines on Airborne Infection Control in Healthcare and other settings” [1]	All areas in the institute are critical, including OPDs, wards, minor OTs, ICUs, laboratories, and postoperative wards	Yes. All types of tuberculosis patients wait in the waiting area	Waiting areas stated to be crucial but not included in the list of critical areas. Information provided as a signed public domain copy by the Public Information Officer designated by the hospital

From the waiting area and infection spread viewpoint, out of the 11 hospitals studied, only three considered waiting areas as critical or high risk. Five of the 11 did not consider waiting areas as critical or high risk. Three hospitals either did not answer the question or did not provide the information (intentionally or taking the cover of the law). It is also important to note that two of the hospitals considered waiting areas as critical but did not list them in the list of critical areas in another question.

## Discussion

India has care centers for the care of human immunodeficiency virus and tuberculosis patients. These healthcare facilities are usually met with huge crowds who gather in the waiting areas [9-11,21]. However, the risk in these facilities is measurable as they are dedicated to known airborne agents such as tuberculosis. The real threat is substantial in general hospitals where there is an intermixing of patients. This leads to high a chance of an unaware suspect getting infected by an equally unaware infected person who may be present in the health facility for the first time and may be non-diagnosed [3]. This is all the more dangerous if the infected individual is an asymptomatic carrier of a disease that is highly transmissible, especially through the airborne route of transmission [22]. The general belief that hospital waiting areas are not very critical may be true in developed countries or high-resource settings that are less crowded [8]. It is worth noting that hospital managers who provided the waiting area information acknowledge the increased threat present in these areas. This is especially apparent because two out of the 11 hospitals stated that waiting areas are critical but did not include waiting areas in the list of critical areas in the hospital. Three hospitals acknowledged the criticality of waiting areas and included it in the list of critical areas in the hospital. The five hospitals that did not consider the waiting area as critical may revisit the same after facing the dire consequences of the COVID-19 pandemic, which has made infection control a reality for all spaces with public interaction, including waiting areas [23,24]. COVID-19 is an airborne infection. Tuberculosis also needs attention as it has not yet been eliminated in India. It is also worth noting that two of the 11 hospitals did not respond to the information request seriously and provided no answer or incomplete answers or evasive answers. This is not only a violation of the Right to Information Act, 2005, but also a lack of compliance with the Public Trust Doctrine. Public hospitals are answerable to the people of the country as their primary accountability. Just like financial information is audited and displayed by government authorities in the public domain, or ambient pollution levels are displayed on roadsides, so should hospitals publicly display the infection control measures on distinguishable and visible display units in public areas. Nosocomial spread of diseases must be brought to light so that steps can be taken to prevent their spread and decrease infectious disease incidence in India and other developing countries.

Including waiting areas in the list of critical areas of the hospitals in infection control manuals and protocols will increase the seriousness of the issue and may lead to a decrease in the spread of nosocomial infections [4]. This will not only be useful in decreasing the load of community spread of tuberculosis but also prepare against the spread of other airborne diseases which include COVID-19, measles, influenza, and severe acute respiratory syndrome coronavirus 1 [25]. After categorizing spaces as critical, hospitals would be able to focus on the implementation of measures aimed at the reduction of airborne spread of diseases in waiting areas. Some steps may include using ultraviolet germicidal irradiation techniques; however, in low-resource settings, dilution ventilation may be appropriate [26-30]. These steps will enable a reduction in the possible spread of airborne infection in waiting areas. The focus of studies in India and other developing countries must be on solutions that are most relevant to the Indian conditions and requirements, especially for matters concerning high population and high crowd density. Indian studies must address the neglected tropical diseases such as tuberculosis, and actions for their prevention must be a primary focus.

## Conclusions

This study set out to find whether the hospitals in Delhi considered waiting areas as critical. The study results have a tilt toward not including waiting areas in the list of high-risk or critical areas in hospitals, with five out of 11 hospitals not considering waiting areas as critical from the infection control viewpoint, especially airborne infection control. Out of the rest, only three considered waiting areas as critical and included them in the list of critical areas in a hospital. These results highlight that waiting areas are not considered critical in hospitals in Delhi. Therefore, it is recommended that waiting areas in all hospitals, regardless of their size or classification, may be considered critical areas from the airborne infection control viewpoint.
